# Ultrastructure Organization of Collagen Fibrils and Proteoglycans of Stingray and Shark Corneal Stroma

**DOI:** 10.1155/2015/686914

**Published:** 2015-06-17

**Authors:** Saud A. Alanazi, Turki Almubrad, Ahmad I. A. AlIbrahim, Adnan A. Khan, Saeed Akhtar

**Affiliations:** Cornea Research Chair, Department of Optometry, College of Applied Medical Sciences, King Saud University, Riyadh, Saudi Arabia

## Abstract

We report here the ultrastructural organization of collagen fibrils (CF) and proteoglycans (PGs) of the corneal stroma of both the stingray and the shark. Three corneas from three stingrays and three corneas from three sharks were processed for electron microscopy. Tissues were embedded in TAAB 031 resin. The corneal stroma of both the stingray and shark consisted of parallel running lamellae of CFs which were decorated with PGs. In the stingray, the mean area of PGs in the posterior stroma was significantly larger than the PGs of the anterior and middle stroma, whereas, in the shark, the mean area of PGs was similar throughout the stroma. The mean area of PGs of the stingray was significantly larger compared to the PGs, mean area of the shark corneal stroma. The CF diameter of the stingray was significantly smaller compared to the CF diameter in the shark. The ultrastructural features of the corneal stroma of both the stingray and the shark were similar to each other except for the CFs and PGs. The PGs in the stingray and shark might be composed of chondroitin sulfate (CS)/dermatan sulfate (DS) PGs and these PGs with sutures might contribute to the nonswelling properties of the cornea of the stingray and shark.

## 1. Introduction

Goldman and Benedek [1] described the structure of shark (spin dogfish),* Squalus acanthias*, and determined the relationship between morphology and nonswelling properties of the cornea. The authors described the morphology of the corneal stroma which consisted of parallel running lamellae which are interconnected across by sutures. A comparative study of collagen in the cornea and sclera of young and adult spiny fish showed that corneal sutural fibrils contained collagen type I, whereas the scleral cartilage matrix contained type II [2]. The immunofluorescence labelling of collagen type I was observed on the thick fibrils but not on the thin fibrils [2]. Praus and Goldman [3] studied the nature of glycosaminoglycans of the shark dogfish and reported that the shark cornea contained 75% galactosaminoglycans of the total glycosaminoglycans present. Recently Scott McCall et al. [4] used the shark dogfish as a model to study collagen cross-linking.

The distribution of corneal layers in the fish cornea varies depending on the aquatic environment. S. P. Collin and H. B. Collin [5], in their review, describe the structure of the cornea in various fish. The Florida gar fish (*Lepisosteus platyrhincus*) cornea consists of the epithelium, BW, stroma, DM, and endothelium. There were aggregates of pigmented granules present in the anterior stroma [6]. The corneas of the sandlance fish (*Limnichthyes fasiciatus*), pipefish (*Corythoichthys paxtoni*), and salamander fish (*Lepidogalaxias salamandroides*) are covered by the epithelium which contains goblet cells. The BW is absent. The stroma is composed of a thin dermal stroma which contained aggregates of pigments and sutures, an iridescent layer, a mucous layer, and anterior sclera stroma. The posterior part of the cornea is covered by the DM and endothelium [7–9].

In ratfish (*Hydrolagus collie*) (cartilaginous fish), the cornea contains the conventional epithelium, BW, stroma with sutures, and DM. The endothelium is not present [10]. The deep-sea teleost [*Coryphaenoides* (*Nematonurus*)* armatus*] contains a very peculiar cornea. The fish cornea consists of an epithelium followed by a thick dermal stroma and mucous layer. The mucous layer is followed by an anterior sclera stroma and iridescent layer. Below that a posterior scleral stroma is present above the DM. The posterior part of the cornea is covered by the endothelium. The BW is absent [11].

The shark (spiny dogfish) and other cartilage fish belong to the* elasmobranchs*. They have the most primitive vertebrate cornea containing CFs organized in stromal lamellae which are crossed by fibrillar sutures. The primitive morphology of the cornea provides its nonswelling properties and maintains transparency [12]. The stingray is also referred to as an* elasmobranch*. There have been no studies carried out on the organization of PGs and CFs of the stingray and shark. In this paper we investigated the ultrastructure of CFs, PGs, and sutures of the shark and stingray stroma. We were interested to see whether there would be any ultrastructural differences between these closely related species.

## 2. Methods

Three stingrays (6-7 year old) (species:* Dasyatis americana*) and three sharks (6-7 year old) (species:* Squalus acanthias*) were used for the study. The eyes were fixed in 4% paraformaldehyde within 60 minutes of the fish's death. The eyes were kept in 4% paraformaldehyde for 12 hours and then washed in phosphate buffer (3 × 15 minutes). One cornea from each animal was taken and was processed for electron microscopy. The corneas were removed from the eyes and were fixed in 2.5% glutaraldehyde containing 0.05% cuprolinic blue (BDH Ltd., Dorset) using a critical electrolyte concentration mode sodium acetate + magnesium chloride buffer overnight at room temperature [13]. The tissue was washed in 2.5% glutaraldehyde in sodium acetate + magnesium chloride (3 × 15 minutes) followed by another wash in sodium tungstate (3 × 15 minutes). The tissue was then dehydrated through a graded ethanol series (50% to 100%) 15 minutes each and 100% acetone (2 × 30 minutes). They were infiltrated in a mixture of acetone and TAAB 031 resin (1 : 1) for 8 hours. The tissue was further infiltrated in 100% TAAB 031 resin for 8 hours (×3). After infiltration into resin, each cornea was cut into 4 quadrates. Each quadrate was placed in green mold for embedding into blocks for sections to be cut. The central part of the cornea in each quadrate was oriented at the cutting surface of the block and polymerized in TAAB 031 resin for 8 hours at 70°C.

All the sections were taken from the central part of the cornea. All the sections were cut at a 90° degree angle to the surface of the cornea (cross section). Semithin and ultrathin sections were cut using an RMC ultracut microtome. Semithin (1 *μ*m) sections were collected on glass slides and stained using toluidine blue. Ultrathin sections were cut from the blocks and collected on 200 mesh copper grids. The sections were stained with 2% uranyl acetate (10 minutes) and lead citrate (10 minutes) and observed using transmission electron microscopy Jeol 1400 (Jeol Ltd., Akishima, Japan). All the digital images were taken from the central part of the cornea. To analyze the CFs and PGs, 3 digital images were taken from the 4 lamellae of the anterior stroma, 4 lamellae from the middle stroma, and 4 lamellae from the posterior stroma above Descemet's membrane.

To observe the PGs distribution without the structure of CF, some sections were not stained with uranyl acetate and lead citrate. The thickness of the lamellae, the* minimum* CF diameter, center-to-center spacing, and PG area were measured using the Soft Imaging System (iTEM, Soft Imaging System GmbH, Münster, Germany) analysis program. The electron micrographs of CFs were processed with the iTEM program and color coded according to the size of the CFs and PGs. The Mann Whitney *U* test (SPSS) was used for statistical analysis because the data were not normally distributed.

### 2.1. Ethical Statement

Tissue procurement and use were in accordance with the Declaration of Helsinki and local regulations. It was ethically approved by the Local Ethical Committee, King Saud University, Saudi Arabia, and King Khalid Eye Specialist Hospital, Riyadh, Saudi Arabia.

## 3. Results

**Figure 1:**
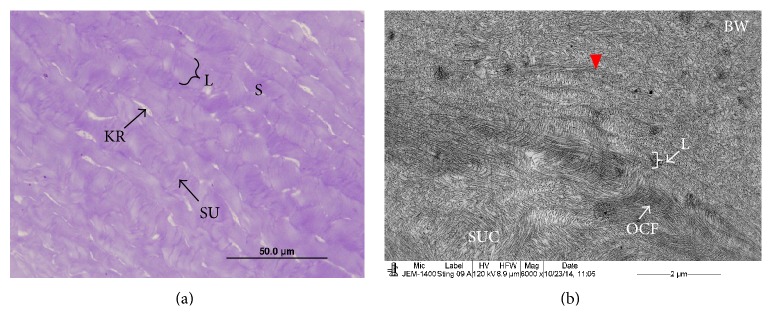
Light and electron micrograph of stingray cornea; (a) middle part of the stroma showing suture running obliquely to the parallel running lamellae; (b) part of the BW and anterior stroma showing BW fibrils blending into the most anterior stromal lamellae, thin lamellae, orthogonal CFs, and orthogonal suture CFs. BW: Bowman's layer, KR: keratocyte, L: lamellae, S: stroma, SU: suture, OCF: orthogonal running CFs, and SUC: orthogonal suture CFs.

**Figure 2:**
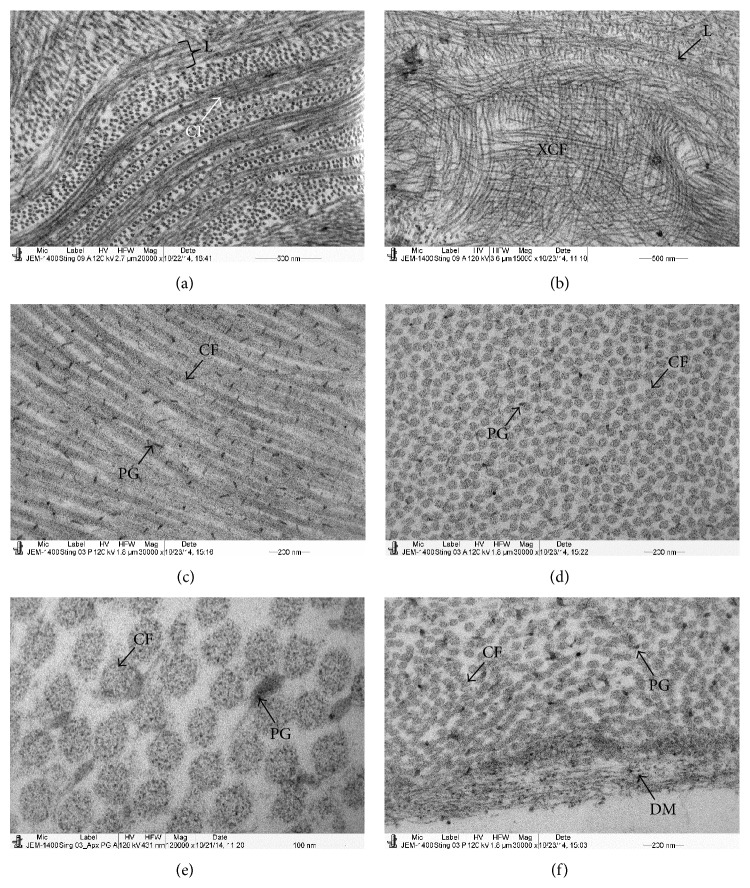
Electron micrograph of collagen fibrils (CF) and proteoglycans (PGs) of stingray cornea; (a) part of the anterior stroma showing thin lamellae containing thin CF (20 nm); (b) part of the anterior stroma showing and orthogonal arrangement of the CFs (XCF); (c) part of the middle stroma showing longitudinally running CFs decorated with PGs; (d) part of the middle stroma showing cross section of uniformly distributed CF and PGs; (e) cross section of CF at high magnification showing microfibrils within CF; (f) posterior part of the stroma showing CF, PGs, and DM composed of microfibrils. BM: basement membrane, BW: Bowman's layer, DM: Descemet's membrane L: lamellae, CF: collagen fibrils, PG: proteoglycans, and XCF: orthogonal arrangement of the CFs.

The corneal stroma of the stingray consisted of the lamellae which were running parallel to the surface of the cornea. The lamellae were crossed by obliquely running sutures (Figure 1(a)). Electron microscopic observations showed that the BW consisted of randomly running CFs (Figure 1(b)). In the posterior part of the BW, the CFs of the BW ran parallel to the corneal surface and blended (arrowhead) into the most anterior stromal lamellae (Figure 1(b)). The anterior lamellae adjacent to the posterior part of the BW were very thin and some of them consisted of three to four CFs (Figures 1(b) and 2(a)). Below these thin lamellae, occasionally, orthogonally running CFs were observed (Figures 1(b) and 2(b)). These fibrils might be entering into the early stages of the formation of sutures. The thickness of the most anterior stromal lamellae was from 0.9 *μ*m to 2.33 *μ*m. The CF diameter in these lamellae was very small (20 nm) (Figure 2(a)). The whole cornea contained approximately 25 lamellae. Under the electron microscope the lamellae thickness varied from the anterior to the posterior stroma. The thinnest lamellae were in the anterior stroma whereas the thickest lamellae were in the middle stroma. The mean thickness of the anterior stromal lamellae (1–9), middle stromal lamellae (10–20), and posterior stromal lamellae (21–26) was 2.62 *μ*m (*n* = 27), 8.65 *μ*m (*n* = 30), and 4.88 *μ*m (*n* = 15), respectively. The lamellae in the anterior stroma were not interlacing but instead were running parallel to each other (Figure 2(b)).

**Figure 3:**
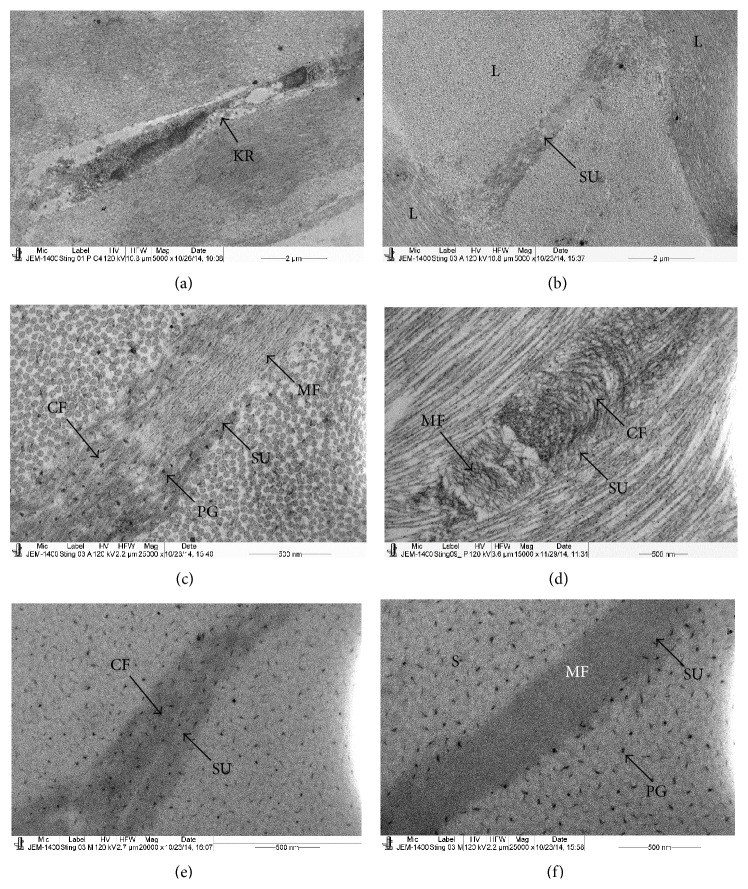
Electron micrograph of stingray fish cornea; (a) part of the stroma showing the keratocyte containing large nucleus; (b) a single suture running obliquely between two lamellae; (c) the middle part of the suture showing presence of CFs and microfibrils. The microfibrils lacked the PGs whereas CFs were decorated with PGs. (d) Sagittal section of a suture showing the CFs surrounding the microfibrils; (e) part of the suture showing presence of PGs on the CFs of the suture; (f) part of suture showing absence of PGs on the microfibrils of suture. CF: collagen fibrils, DM: Descemet's membrane, L: lamellae, KR: keratocyte, MF: microfibrils, PG: proteoglycans, and SU: suture.

The CFs within the lamellae in the anterior, middle, and posterior stroma were running parallel to each other. These parallel running CFs were decorated with PG filaments (Figure 2(c)). The cross section of the CFs showed the distribution the CFs of variable diameters within the lamellae (Figure 2(d)). There were electron dense microfibrils present within the CFs (Figure 2(e)). The posterior part of the cornea was covered by the DM (Figure 2(f)). The DM consisted of very fine, loosely interwoven fibrils (Figure 2(f)). The keratocytes were distributed throughout the stroma and contained a large nucleus (Figure 3(a)).

Throughout the stroma of the stingray, sutures were observed running obliquely to the lamellae (Figure 3(b)). In some places two or more sutures were running in a group across the lamellae. The longitudinal section of the sutures showed that they consisted of two types of fibrils, thick CFs and thin microfibrils (Figure 3(c)). The cross section of the suture showed that the thin microfibrils were surrounded by the thick CFs (Figure 3(d)). The CFs were decorated with PGs (Figure 3(e)). The attachment of the PGs was not observed within the microfibrils (Figure 3(f)).

**Figure 4:**
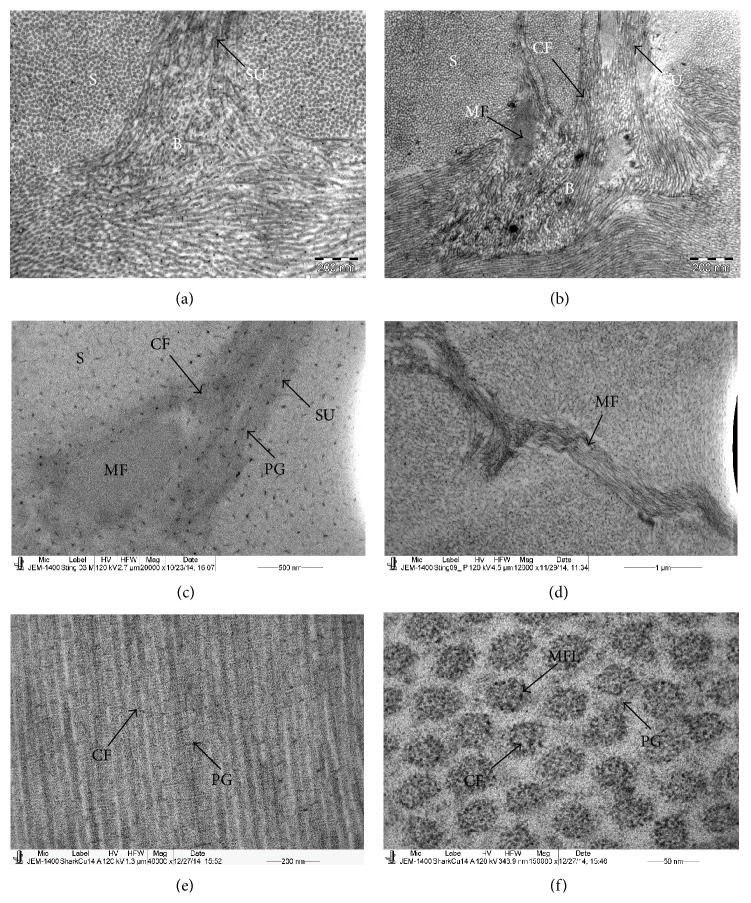
Electron micrograph of stingray cornea; (a) posterior part of a single suture containing randomly running CFs embedded within the lamella. (b) Basal part of multiple sutures containing randomly running CF which were embedded within the lamella. Group of microfibrils were present among the CF. (c) Posterior part of the suture showing presence of PGs on the CF but not on the microfibrils; (d) longitudinally running microfibrils of suture not surrounded by CF; (e) electron micrograph of longitudinally running CF of the middle stroma of shark; (f) electron micrograph of cross section of CF of the middle stroma of shark. CF: collagen fibrils, L: lamellae, MFL: microfilament, PG: proteoglycans, S: stroma, and SU: suture.

The CFs in the anterior and posterior part of the suture were randomly arranged and blended with the CFs of the lamellae (Figure 4(a)). In some places the three sutures were aggregated at the same place (Figure 4(b)). The anterior and posterior end of the sutures also contained groups of microfibrils surrounded by the CFs which blended into the lamella (Figures 4(a) and 4(b)). At the anterior and posterior end of the sutures, PGs were attached to the CFs but not to the microfibrils (Figure 4(c)). The longitudinally running microfilaments were occasionally observed in the stroma (Figure 4(a)).

The structure of the shark cornea was very similar to the structure of the stingray cornea except for the distribution of PGs around the CFs. In the shark, the sutures of the three or four lamellae were connected to each other and ran in straight lines unlike in the stingray. The PGs were small in size and closely arranged around the CFs (Figure 4(e)). The CFs contained very prominent microfilaments (Figure 4(f)).

### 3.1. Collagen Fibril Diameter and Interfibrillar Spacing

**Figure 5:**
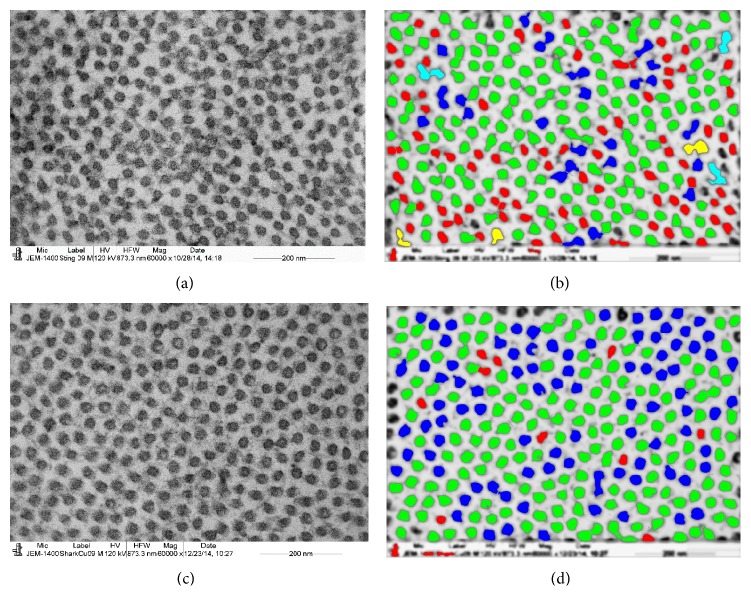
Electron micrograph and colour coded digital images of CF of the middle stroma of stingray and shark; (a) electron micrograph of middle stroma of stingray; (b) colour coded digital image of (a); (c) electron micrograph of middle stroma of stingray; (d) colour coded digital image of (c). Please note that, in stingray, most of the CFs were in the range from 15 to 25 nm (red and green) and few of them were in the range from 25 to 30 nm (blue). In shark very few CFs were in the range from 15 to 20 nm, and most of the CFs were in the range from 20 to 30 nm (green and blue). Blue: 25–30 nm; yellow: 30–35 nm; terracotta: 35–40 nm; pink: 40–45 nm.

The CF diameter of the stingray and shark was analyzed by processing with an electron micrograph (Figures 5(a) and 5(c)) using the iTEM program. After processing, the images were displayed using color coding to demonstrate the distribution of variable diameters of CFs (Figures 5(b) and 5(d)). In the stingray, the mean diameters of the CFs in the anterior (22.06 ± 3.22 nm, *n* = 263), middle (22.12 ± 4.40 nm, *n* = 255), and posterior stroma (22.21 ± 5.05, *n* = 307) were very similar to each other (Table 1). The interfibrillar spacing between the CF in the anterior stroma (32.74 ± 8.19 nm, *n* = 263) was less than the interfibrillar spacing in the middle stroma (36.9 ± 5.12, *n* = 255) and posterior stroma (37.83 ± 7.48 nm, *n* = 307) (Table 1). In the shark, the CF diameters in the anterior (23.37 ± 2.92 nm, *n* = 278), middle (24.80 ± 2.51 nm, *n* = 263), and posterior stroma (24.59 ± 2.44 nm, 303) were also very similar to each other.

In the shark the interfibrillar spacing in the anterior (35.84 ± 4.18 nm, *n* = 278) and posterior stroma (35.17 ± 4.37 nm, *n* = 263) was less than in the middle stroma (37.86 ± 3.93 nm, *n* = 303). In the stingray, most of the CFs were in the range from 15 to 25 nm (red and green) and a few of them were in the range from 25 to 30 nm (blue). In the shark a very few CFs were in the range from 15 to 20 nm (red and green), and most of the CFs were in the range from 20 to 30 nm (green and blue). The CF diameter of the stingray in the anterior, middle, and posterior stroma was significantly less (*p* < 0.0001) than those in the shark (Table 1). The interfibrillar spacing in the anterior, middle, and posterior stroma of the stingray was also significantly different (*p* < 0.0001) from those of the shark (Table 1).

### 3.2. PG Mean Area

**Figure 6:**
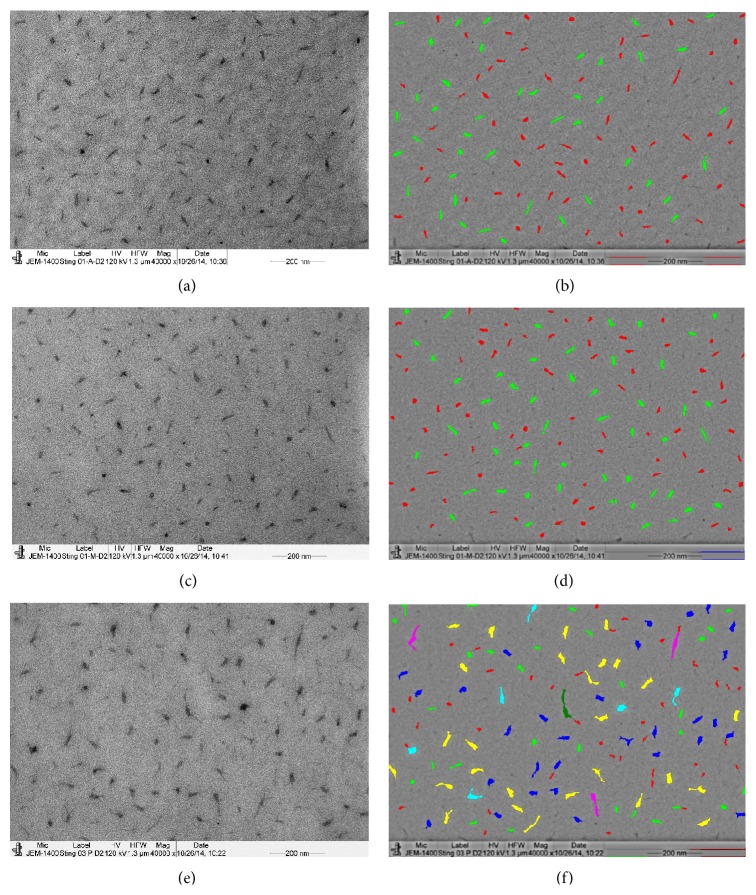
Electron micrograph and colour coded digital images of the PGs of stingray. The electron micrograph was taken from section of corneal stroma that was not stained with uranyl acetate and lead citrate; (a) electron micrograph of PGs of the anterior stroma; (b) colour coded digital image of (a); (c) electron micrograph of PGs of the middle stroma; (d) colour coded digital image of (c); (e) electron micrograph of PGs of the posterior stroma. Please note that PGs are star shaped; (f) colour coded digital image of (e). Red: 50–257, green: 257–464, blue: 464–671, yellow: 671–878, terracotta: 878–1045, pink: 1085–1292, and brown: 1292–1500.

In the stingray, the different sizes of PGs were decorated around the CFs of the stroma (Figures 2(d), 2(e), and 2(f)) and around the CFs of the suture (Figures 3(d) and 3(e)). The structure of the PGs in the anterior, middle, and posterior stroma was identified in the electron micrographs taken from the corneal sections which were not stained with uranyl acetate and lead citrate (Figures 6(a), 6(c), and 6(e)). The electron micrographs (Figures 6(a), 6(c), and 6(e)) were processed using the iTEM program and the variable sizes of the PGs were demonstrated by color coding in the digital images (Figures 6(b), 6(d), and 6(f)). The PGs in the anterior and middle stroma were rod shaped and their sizes ranged from 50 to 464 nm^2^ (red and green color) (Figures 6(b) and 6(d)). The PGs in the posterior stroma were very large and ranged from 465 to 1292 nm^2^ (blue, yellow, and pink) (Figure 6(f)). The PGs in the posterior stroma were thick in the middle and thin at the edges. Some of them were star shaped; others were rod shaped. The mean area of the PGs is shown in Table 2. The mean PGs areas in the anterior and middle stroma were similar to each other and not significantly different. The PGs area in the posterior stroma (324.64 ± 182.00 nm, *n* = 465) was significantly (*p* < 0.001) higher compared to the mean PGs area of the anterior (224.54 ± 82.64 nm, *n* = 384) and middle (225.52 ± 80.86 nm, *n* = 460) stroma. The density of the PGs was higher in the middle and posterior stroma compared to the anterior stroma in the stingray (Table 2).

**Figure 7:**
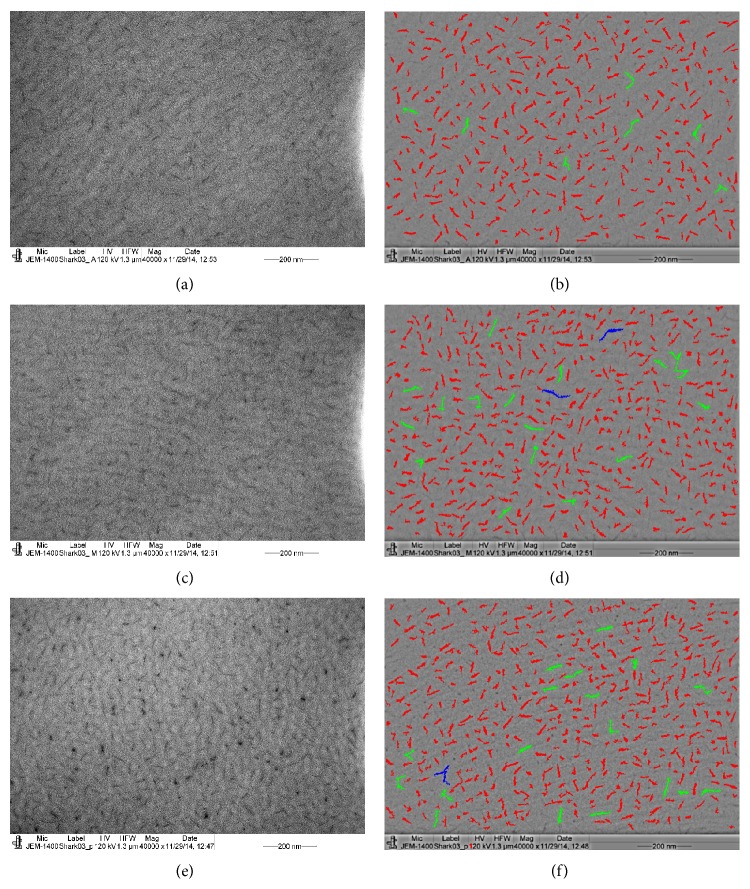
Electron micrograph and colour coded digital images of the PGs of shark. The electron micrograph was taken from section of corneal stroma that was not stained with uranyl acetate and lead citrate; (a) electron micrograph of PGs of the anterior stroma; (b) colour coded digital image of (a); (c) electron micrograph of PGs of the middle stroma; (d) colour coded digital image of (c); (e) electron micrograph of PGs of the posterior stroma; (f) colour coded digital image of (e). Red: 50–257, green: 257–464, blue: 464–671, yellow: 671–878, terracotta: 878–1045, pink: 1085–1292, and brown: 1292–1500.

The PGs in the shark were analysed as described above. The electron micrographs of the PGs in the anterior, middle, and posterior stroma, shown in Figures 7(a), 7(c), and 7(e), were color coded according to their variable sizes and are shown in Figures 7(b), 7(d), and 7(f). The mean area size of the PGs in the anterior stroma (194.52 ± 79.26 nm) was less than those in the middle (209.09 ± 92.04 nm) and posterior (201.65 ± 92.67 nm) stroma (Table 2). The density of the PGs was also less in the anterior stroma than in the middle and posterior stroma (Table 2). The spacing between the PGs was similar in all three regions.

The PGs analysis also showed that the mean PGs area of the anterior (*p* < 0.001), middle (*p* < 0.003), and posterior (*p* < 0.001) stroma of the stingray was significantly higher compared to the mean PGs area of the anterior, middle, and posterior stroma of the shark. The density of the PGs in the stingray in the anterior, middle, and posterior stroma was less than those in the shark stroma (Table 2). The spacing between the PGs in the shark was also significantly (*p* < 0.001) less compared to the spacing between the PGs of the stingray (Table 2).

## 4. Discussion

The cornea is the outermost transparent layer of the eye. The normal human cornea is composed of five layers, epithelium, Bowman's layer (BW), stroma, Descemet's membrane (DM), and endothelium [14]. The stroma constitutes a major part of the cornea. It is composed of CFs lamellae which contain angular lamellae relative to the transverse lamellae (fibres) along the corneal surface [15]. These lamellae (fibres) branch several times and connect to each other and in many cases interleave into the Bowman's layer [15]. The highest degree of this interconnectivity was observed in the transverse lamellae in the anterior stroma [15]. It is believed that transverse lamellae and their interconnectivity play an important role in determining corneal biomechanics and stress distribution, resisting the formation of regions with stress, which are more susceptible to deformation [15]. It was also suggested that interconnectivity of the lamellae in the anterior stroma is likely to serve to stabilize corneal shape and prevent lamellar slippage [15, 16]. The interconnectivity also provides rigidity to the anterior stroma and specifies corneal curvature and shape [17].

Our studies showed that the stroma of the stingray consisted of concentric lamellae lying in parallel to the corneal surface and interconnected across by sutures throughout the stroma. The structure of the stingray cornea was very similar to the structure of shark cornea described by Goldman and Benedek [1]. The interconnectivity of lamellae by sutures in the stingray and shark can be compared with the interconnectivity of lamellae in the anterior human stroma described by Winkler et al. [15]. We believe that interconnectivity of the lamellae by sutures in the stingray and shark plays an important role in determining corneal biomechanics and stabilizing corneal shape, as has been observed in the anterior stroma of the human cornea by Winkler et al. [15]. This phenomenon might inhibit stromal swelling in the stingray and shark.

Sutures are an important and characteristic feature of the shark and stingray. These sutures were also observed in the stroma of salamander and shark fish [1, 9]. The sutures in the shark run in straight lines continuously across 3 or 4 lamellae and are connected to each other. In the stingray the sutures of different lamellae were not connected to each other and did not run in straight lines. Our observations showed that the sutures in the stingray and shark consisted of thick CFs and thin microfibrils. The thin filaments were surrounded by thick fibrils as previously reported by Goldman and Benedek [1]. We are uncertain about the nature of microfibrils. Thick CFs were composed of collagen type I whereas the thin fibrils were not composed of collagen type I [2]. The thin fibrils were never twisted whereas the thick fibrils were observed twisting and joining together [1]. The thick fibrils of the sutures were decorated by PGs but not the thin fibrils.

Goldman and Benedek [1] reported that thin fibrils never stretched within the sutures and they inhibit the stretching of the sutures. It has been suggested that the cylindrical core of thin fibrils was the principal bundle of the suture [1]. We presumed that the cylindrical core of thin fibrils provides mechanical strength and resistance to swelling of the cornea. If the stretching of sutural fibrils was inhibited then the thickness of the cornea might be limited by the length of the thin fibril bundles. Occasionally orthogonally running CFs were observed suggesting that the fibrils might be entering into the early stages of the formation of a suture and blending with the lamellar CFs. This phenomenon might be providing mechanical strength to the lamellar CFs and might be restricting the separation of the CFs within the lamellae and the separation of the lamellae from each other [1].

In the cornea, three core proteins (lumican, keratocan, and mimecan) bearing glycosaminoglycan (GAG) chains of keratan sulfate and one protein (decorin) bearing GAG chains of chondroitin/dermatan sulfate are present [18]. It has been reported that lumican plays an important role in regulating the CF diameter and it was higher in concentration in the posterior part of the stroma compared with that in the anterior stroma [19]. The CF diameters in the anterior, middle, and posterior stroma were not significantly different. The density of the CFs was higher in the posterior stroma. In the shark, the CF diameter and density were significantly smaller compared with those in the middle and posterior stroma. This could be due to higher concentrations of lumican in the posterior stroma. Comparing the shark and stingray, the CF diameters of the stingray were significantly smaller compared to the CF diameters of the shark throughout the stroma. This could also be due to the reduced amount of lumican present in the stingray compared with that in the shark.

Goldman and Benedek [1] reported that in the shark the CFs diameters in the anterior stroma (32.7 ± 2.1 nm) were larger than in the posterior stroma (28.2 ± 3.8 nm). In the present study, the means of minimum CF diameter of shark in the anterior (23.37 ± 2.92 nm), middle (24.80 ± 2.51 nm), and posterior (24.59 ± 2.44 nm) stroma were very similar to each other. The mean of minimum CFs diameters of the shark in our study was smaller than the CFs diameter of the shark reported by Goldman and Benedek [1]. This could be due to different processing techniques or methods of calculating the diameter. In our experiments tissue was processed with glutaraldehyde containing cuprolinic blue in sodium acetate + magnesium buffer and TAAB 031 resin was used, whereas they processed tissue in glutaraldehyde and osmium tetroxide embedded in Epon 812. Goldman and Benedek [1] reported that the density of CFs from anterior to posterior stroma of the shark was approximately in the range from 450/*μ*m to 540/*μ*m whereas our studies showed that the density of CFs from anterior stroma to posterior stroma was in the range of 547/*μ*m to 595/*μ*m.

Scott [20] measured the PGs of bovine cornea with electron microscopy. He reported that stained CS/DS filaments are ~70 nm long, whereas KS PGs are ~70 nm long. Lewis et al. [21] reported that PGs composed of CS/DS were very large (about 300 nm) whereas PGs composed of KS were small (about 65 nm). In the stingray, the PGs in the anterior and middle stroma were small and rod shaped. It is believed that these large PGs in the posterior stroma of stingray are composed of CS/DS PGs. The presence of the large PGs in the posterior stroma may play an important role in regulating the hydration of the stingray cornea.

The PGs analysis in the shark showed that there was no significant difference in the mean area of the PGs in the anterior, middle, and posterior stroma. The density of PGs in the posterior and middle stroma was significantly (*p* < 0.001) higher compared to the density of the PGs in the anterior stroma. It is surprising that both the stingray and the shark belong to the same class, but the PGs mean areas in the stingray were significantly (*p* < 0.001) larger compared to the PGs mean area in the shark in the anterior, middle, and posterior stroma. The density of the PGs however was significantly less in the stingray compared to the PGs density in the shark in all regions (anterior, middle, and posterior stroma). PGs of stingray and shark, like those in bovine and mouse, show no specific order [22]. The distance between the PGs in the shark is significantly smaller compared to those in the stingray. It could be possible that this close association of the high density of PGs in the shark may strengthen the stromal CFs which provide strength to their large sized cornea as previous studies showed that large animals such as camels have a large density of small PGs [23]. It has been suggested that in comparison with the cornea of more advanced vertebrates, the nonswelling shark cornea contains large amounts of highly-sulfated “chondroitin sulfate” and small amounts of “keratan sulfate” which regulate hydration in the aquatic environment [3].

Scott McCall et al. [4] suggested that interlamellar bonds increase the mechanical strength of the cornea because they could physically link the entire adjacent lamellae of the corneal stroma. These physical interactions could occur between separate collagen fibrils both within an individual lamella and in adjacent lamellae [17]. These interactions between the CFs could arise via the terminal domains of the GAG chains that extend from the sides of all collagen fibrils. In elasmobranch corneas, the sutures provide a physical interlamellar bond supporting the mechanical strength of the cornea and inhibiting the swelling of the cornea in the shark [1, 24].

We suggest that the physical strength of the sutures might be provided by the interaction of the CFs which occurs via the terminal domain of the GAG chains that extend from the side of the CFs. We believe that PGs play an important role in providing the physical strength of sutures that inhibit the swelling of the cornea in the stingray and shark. The presence of high levels of chondroitin sulfate and low levels of keratan sulfate in the shark [10] and in the stingray may also contribute to the nonswelling properties of their corneas. Further studies are required to investigate the role of keratan sulfate and chondroitin sulfate by using their specific antibodies and employing biochemistry, immunohistochemistry, and immunogold techniques.

## Figures and Tables

**Table 1:** Mean collagen fibril diameter of anterior, middle, and posterior stroma of stingray and shark cornea.

	Density/*μ*m		Minimum mean diameter ± standard error (nm)		Mean interfibrillar spacing ± standard error (nm)	
	Stingray	Shark	Stingray	Shark	Stingray	Shark
Anterior stroma	516	547	22.06 ± 0.31^*∗*^	23.37 ± 0.23^*∗*^	32.74 ± 0.53^*∗∗*^	35.84 ± 0.34^*∗∗*^
Middle stroma	444	516	22.12 ± 0.27^*∗*^	24.80 ± 0.16^*∗*^	36.9 ± 0.32^*∗∗*^	37.86 ± 0.24^*∗∗*^
Posterior stroma	603	595	22.21 ± 0.28^*∗*^	24.59 ± 0.12^*∗*^	37.83 ± 0.28^*∗∗*^	35.17 ± 0.23^*∗∗*^

Value denotes ± standard error.

^*∗*^ *p* < 0.0001.

^*∗∗*^ *p* < 0.0001.

**Table 2:** Mean area of proteoglycans of anterior, middle, and posterior stroma of stingray and shark.

	Density		Mean Area (nm^2^)		Spacing	
	Sting-ray	Shark	Sting ray	Shark	Stingray	Shark
Anterior stroma	131	384	225.52 ± 5.04^*∗*^	194.52 ± 4.04^*∗*^	64.33 ± 1.44^†^	37.64 ± 0.46^†^
Middle stroma	123	460	224.54 ± 5.25^*∗∗*^	209.09 ± 4.29^*∗∗*^	70.01 ± 1.22^†^	34.55 ± 0.38^†^
Posterior stroma	135	465	324.64 ± 11.09^*∗*^	201.65 ± 4.29^*∗*^	64.14 ± 1.18^†^	33.79 ± 0.42^†^

^*∗*^ *p* < 0.0001.

^*∗∗*^ *p* < 0.003.

^†^ *p* < 0.0001.
